# Transcriptome and Metabolome Analyses of *Taxus chinensis* var. *mairei* Tissues Provide New Insights into the Regulation of Paclitaxel Biosynthesis

**DOI:** 10.3390/plants14121775

**Published:** 2025-06-10

**Authors:** Luyuan Jiang, Yanyan Li, Xiaoyang Jiang, Fenjuan Shao, Wenli Wu, Fan Xu, Iain Wilson, Angela Hoffman, Yanfang Yang, Deyou Qiu

**Affiliations:** 1 State Key Laboratory of Tree Genetics and Breeding, Key Laboratory of Tree Breeding and Cultivation of National Forestry and Grassland Administration, Research Institute of Forestry, Chinese Academy of Forestry, Beijing 100091, China; bachelorjly@163.com (L.J.); xiaoyang9612@126.com (X.J.); shaofenjuan@caf.ac.cn (F.S.); wwlarroyo@163.com (W.W.); xufan09@caf.ac.cn (F.X.); echoyyf@caf.ac.cn (Y.Y.); 2 College of Medicine, Pingdingshan University, Pingdingsha 467000, China; liyanyan041@163.com; 3 CSIRO Agriculture and Food, Canberra, ACT 2601, Australia; 4 Department of Natural Sciences, Saint Martin’s University, Lacey, WA 98503, USA; ahoffman@stmartin.edu

**Keywords:** *Taxus chinensis* var. *mairei*, transcriptome, metabolome, paclitaxel biosynthesis, regulation

## Abstract

*Taxus* is the natural source of the anticancer drug paclitaxel. Although significant progress has been made in elucidating the biosynthetic pathway of paclitaxel, its tissue-specific accumulation and associated regulatory networks in *Taxus* remains unclear. In this study, we conducted integrated transcriptomic and metabolomic analyses of the root, leaf, shoot, bark, and wood of *Taxus chinensis* var. *mairei* to investigate the tissue-specific biosynthesis and accumulation of paclitaxel. We found that paclitaxel, 10-deacetylbaccatin III, and several taxoids were significantly enriched in the leaf, bark, and shoot, while paclitaxel derivatives, such as taxayunnansin A and taxol B, accumulated primarily in the root. Most genes involved in paclitaxel biosynthesis showed the highest expression in the root and the lowest in the wood. Using weighted gene co-expression network analysis (WGCNA), we identified several candidate transcription factors potentially regulating paclitaxel biosynthesis. Further validation using yeast one-hybrid (Y1H) and dual-luciferase reporter assays confirmed that ERF68 activates the expression of *taxane-2α-hydroxylase* (T2H) gene, a key gene in the paclitaxel biosynthesis pathway. Collectively, our finding provides crucial insights into the transcriptional regulation of paclitaxel biosynthesis in *Taxus*.

## 1. Introduction

*Taxus* spp. (Taxaceae) are evergreen trees or shrubs, predominantly distributed in the northern hemisphere [1]. These species produce a variety of bioactive compounds, including paclitaxel (also named taxol), polysaccharides and flavonoids [2,3,4]. As a vital anticancer drug, paclitaxel was firstly isolated from the bark of *T. brevifolia* Nutt., and its structure was elucidated by Wall and Wani in 1971 [2]. Its anticancer mechanism involves the disruption of cancer cell mitosis [5], and it has been widely used in the treatment of ovarian, breast, esophageal, lung, and stomach cancers [6]. Due to the slow growth and rarity of many *Taxus* species, coupled with the extremely low natural yield of paclitaxel, paclitaxel production cannot meet the global demand. Intermediates such as 10-deacetylbaccatin III (10-DAB) and baccatin Ⅲ are extracted from the branches and needles of *Taxus* and used for the semi-synthesis of paclitaxel [7]. Synthetic biology and metabolic engineering approaches have newly emerged as promising alternatives to explore paclitaxel biosynthesis in recent years [8,9,10,11,12,13].

Paclitaxel is a complex diterpene compound synthesized through a network involving both terpene and phenylalanine pathways. With the development of the sequencing technology, the publications of three sets of *Taxus* chromosome-level genomes have provided new gene resources for the studies of the paclitaxel biosynthesis and its regulation in *Taxus* [14,15,16]. The biosynthetic pathway of paclitaxel can be divided into three main stages, including the synthesis of taxa-4(5), 11(12)-diene as a taxane skeleton, the synthesis of intermediate baccatin III, and the assembly of the side chain. The universal precursors of terpenoids, dimethylallyl diphosphate (DMAPP) and isoprenyl diphosphate (IPP), are produced by the mevalonic pathway (MVA) in the cytosol or via the methylerythritol phosphate (MEP) pathway in plastids [17,18]. Geranylgeranyl diphosphate (GGPP), which is synthesized from three IPP molecules and one DMAPP by GGPP synthase (GGPPs), serves as a precursor for paclitaxel [19]. GGPP is then catalyzed by taxadiene synthase (TS) to form taxa-4(5),11(12)-diene and taxa-4(20),11(12)-diene [20]. In the second stage, taxa-4(5),11(12)-diene is hydroxylated at C1,C2, C5, C7, C9, C10, and C13 by taxane-1β-hydroxylase (T1H), taxane-2α-hydroxylase (T2H), taxadiene-5α-hydroxylase (T5H), taxane-7β-hydroxylase (T7H), taxane-9α-hydroxylase (T9H), taxane-10β-hydroxylase (T10H), and taxane-13α-hydroxylase (T13H); acylated at C5 and C10 by taxadiene-5α-ol-O-acetyl transferase (TAT) and 10-deacetyl baccatin III-10β-O-acetyltransferase (DBAT); epoxidized at C4 and C20 by epoxidase or oxetanized by taxaneoxetanase (TOT); oxidized at C9 by taxane-9α-dioxygenase (T9O); and modified at C2 by taxane-2α-O-benzoyltransferase (TBT) to produce the key intermediate baccatin III [21,22,23,24,25,26,27,28,29,30]. In the third stage, the side chain derived from α-phenylalanine is synthesized via phenylalanine aminomutase (PAM) and β-phenylalanine coenzyme A ligase (PCL). Baccatin III-3-amino-3-phenylpropanoyltransferase (BAPT) catalyzed the conjugation of the β-phenylalanoyl-CoA side chain to baccatin III, forming 3′-N-debenzoyl-2′-deoxytaxol. Then, 3′-N-debenzoyl-2′-deoxytaxol is hydroxylated by taxane 2′α-hydroxylase (T2′H). Finally, 3′-N-debenzoyl-taxol was modified by 3′-N-debenzoyl-2′-deoxytaxol-N-benzoyltransferase (DBTNBT), yielding the end product, paclitaxel [31,32,33,34,35]. Due to the complex biosynthetic route and various branching reactions, over 600 taxoids have been isolated from *Taxus* species to date [36]. Some of these derivatives, like cephalomannine and 7-epi-taxol, share the same antitumor mechanism and can be converted into paclitaxel, representing potential alternative sources of paclitaxel that may help alleviate supply limitations [37,38,39,40].

Although the paclitaxel biosynthesis genes were almost thoroughly studied, some regulators remain unknown. Previous studies have shown that transcription factors (TFs) play significant roles in the regulation of paclitaxel biosynthesis by activating or repressing the expression of structural genes in the paclitaxel biosynthesis pathway. These TFs are often hormone-inducible or tissue/sex-specific. For example, jasmonate-responsive ERFs, such as TcERF12 and TcERF15, act as the repressor and activator of TS, respectively, by binding to the GCC-box in its promoter [41]. TcMYC2a positively regulates the expression of *TS* and *TcERF15* [42]. TcWRKY1 was also induced by MeJA and can activate the transcription of *DBAT* [43]. Salicylic acid-responsive factor TcWRKY33 promoted the expression of *DBAT* and *TcERF15* [44]. TcMYB29a, an abscisic acid-responsive factor, upregulated *T5H* [45]. Phloem-specific TmMYB3 positively regulates paclitaxel biosynthesis by activating the expression of *TBT* and *TS* [46]. It has been reported that paclitaxel accumulates at a significantly higher level in female *T. media*. The female-predominant MYB39-bHLH13 complex trans-activates the expression of *GGPPS* and *T10H* genes [47]. Endodermal cell-specific MYB47 significantly activates *T5H* expression in *T. mairei* stems. Xylem parenchyma cell-specific bHLH68 significantly activates *TAT* and *T10H* expression [48]. MYB17 and bHLH46 significantly inhibit the expression of *TS* and *GGPPS* in *T. mairei* leaves, respectively [49]. A regulatory network, including 10 enzyme genes of the paclitaxel pathway and 28 TFs, was reported [50]. However, the TFs involved in the regulation of the expression of the paclitaxel genes in different *Taxus* tissues require further investigation.

In this study, we performed a comprehensive analysis combining the metabolomic profiling and RNA sequencing of five tissues (root, leaf, shoot, bark, and wood) of *T. chinensis* var. *mairei* to investigate tissue-specific paclitaxel accumulation. We identified differentially expressed genes (DEGs) and differentially accumulated metabolites (DAMs) associated with paclitaxel biosynthesis. Through weighted gene co-expression network analysis (WGCNA), we constructed a regulatory network for paclitaxel accumulation and identified ERF68 as a potential regulator of the biosynthetic pathway. Y1H and dual-luciferase reporter assays demonstrated that ERF68 activates the expression of a key paclitaxel biosynthesis gene, *T2H*. Together, our findings reveal a novel mechanism underlying the regulation of paclitaxel biosynthesis mediated by TF ERF68.

## 2. Results

### 2.1. Overview of the Metabolomes

**Figure 1:**
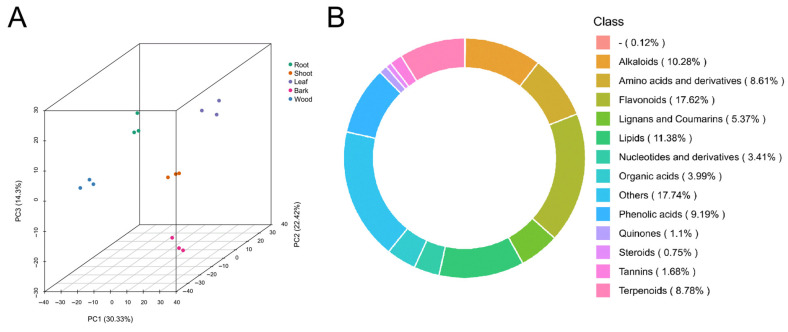
Summary of metabolome. (**A**) The principal component analysis of metabolome data from the 15 samples. (**B**) Class-count ring of metabolome data. “-” represents unknown metabolites.

To investigate variations in metabolite accumulation between different tissues, a widely targeted metabolomic analysis was conducted. A total of 1731 metabolites were identified across five different tissues (Table S1). The total ion chromatograms (TICs) of the mixed-sample extracts are shown in Figure S1. Principal component analysis (PCA) was performed to assess the overall metabolic variation between samples. The first two principal components, PC1 and PC2, explained 30.33% and 22.42% of the total variance, respectively. The PCA result shows five distinct sample groupings, indicating clear metabolic differentiation between the tissues (Figure 1A). The identified 1731 metabolites were assigned to 14 categories, including flavonoids (17.62%), lipids (11.38%), alkaloids (10.28%), phenolic acids (9.19%), terpenoids (8.78%), amino acids and derivatives (8.61%), lignans and coumarins (5.37%), organic acids (3.99%), nucleotides and derivatives (3.41%), tannins (1.68%), quinones (1.1%), steroids (0.75%), others (17.74%), and uncategorized metabolites (0.12%) (Figure 1B). The results show the diversity of the metabolites in five tissues.

### 2.2. Analysis of DAMs in Different Tissues

**Figure 2:**
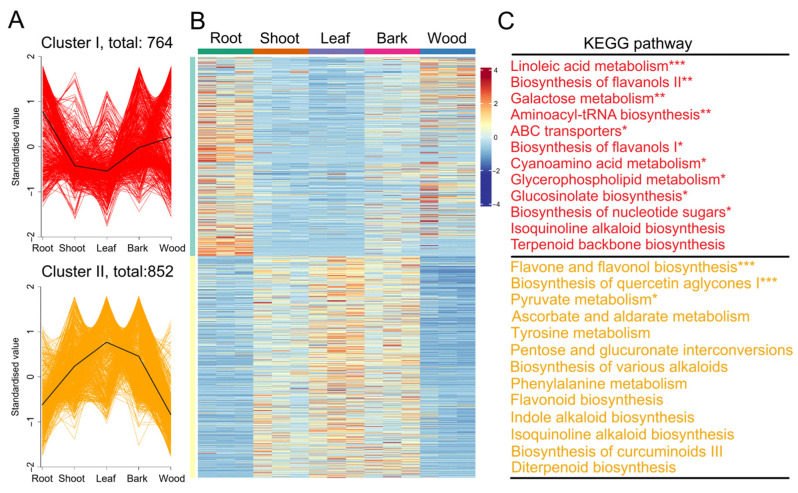
The differentially accumulated metabolites (DAMs) among five tissues. (**A**) K-means analysis of DAMs. (**B**) A heatmap of DAMs. (**C**) Kyoto Encyclopedia of Genes and Genomes enrichment analysis of DAMs. “*” indicates a significant difference of *p* < 0.05. “**” indicates a significant difference of *p* < 0.01. “***” indicates a significant difference of *p* < 0.001.

A total of 1616 DAMs from different comparisons (root/leaf, wood/leaf, bark/leaf, root/shoot, wood/root, wood/shoot, bark/wood, bark/root, shoot/leaf, and bark/shoot) were classified into two distinct clusters (Figure 2). Clusters I comprised 764 metabolites that predominately accumulated in the root and wood tissue, whereas Cluster II (852 metabolites) were enriched in the leaves, shoot, and bark tissues. The Kyoto Encyclopedia of Genes and Genomes (KEGG) analysis revealed that the significantly enriched pathways in Cluster I included ‘linoleic acid metabolism’, ‘biosynthesis of flavanols II’, ‘galactose metabolism, aminoacyl-tRNA biosynthesis’, ‘ABC transporters’, ‘biosynthesis of flavanols I’, ‘cyanoamino acid metabolism’, ‘glycerophospholipid metabolism’, ‘glucosinolate biosynthesis’, and ‘biosynthesis of nucleotide sugars’ (Figure 2). In contrast, Cluster II was significantly enriched in ‘flavone and flavonol biosynthesis’, ‘biosynthesis of quercetin aglycones I’, and ‘pyruvate metabolism’. Interestingly, Cluster I contained dimethylallyl diphosphate (DMAPP), a key intermediate in the terpenoid backbone biosynthesis pathway that provides essential precursors for paclitaxel biosynthesis. Meanwhile, Cluster II included 10-deacetylbaccatin III and paclitaxel, both of which are critical intermediates or end products in the diterpenoid biosynthesis pathway.

### 2.3. Overview of the Transcriptomes

**Figure 3:**
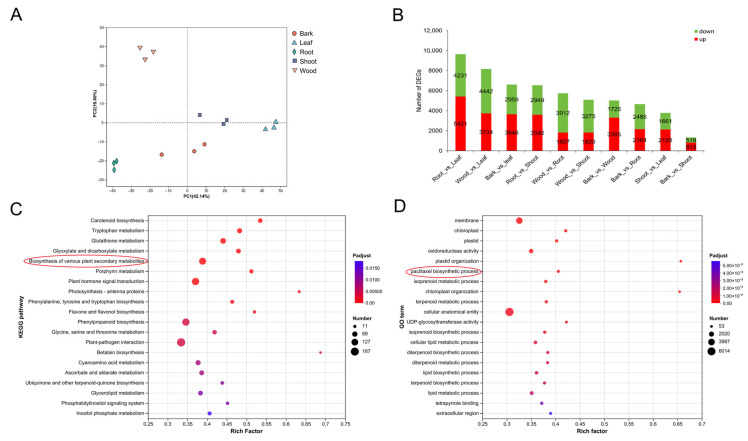
Analysis of differentially expressed genes (DEGs). (**A**) PCA of transcriptome data from the 15 samples. (**B**) The statistics of up- and down-regulated DEGs in the different comparisons. (**C**) Kyoto Encyclopedia of Genes and Genomes enrichment analysis of DEGs. (**D**) Gene Ontology enrichment analysis of DEGs.

To investigate the transcription regulation mechanisms of different tissues, RNA sequencing was performed on the same samples used for metabolomic analysis. Each sample generated on average 6.01 Gb of data, and a total of 105.66 Gb clean data was obtained. The Q30 values were above 96.6%, indicating the sequencing data were reliable (Table S2). Clean reads from each sample were aligned with the *T. wallichiana* genome [14], with the mapping rate ranging from 86.39% to 90.39% (Table S3). In total, 42,657 expressed genes were detected. Among them, 32,631 genes were successfully annotated through the BLAST alignment against six public databases. Specifically, 21,950 (67.27%) genes were annotated in the Gene Ontology (GO) database, 14,034 (43.01%) in KEGG, 28,678 (87.89%) in EggNOG, 31,944 (97.89%) in NR, 25,806 (79.08%) in Swiss-Prot, and 26,098 (79.98%) in Pfam (Figure S2). Principal component analysis (PCA) revealed that the three biological replicates from each tissue group clustered closely together, indicating good reproducibility and reliability of the transcriptomic data (Figure 3A).

### 2.4. Analysis of DEGs in Different Tissues

Based on transcripts per million (TPM) values, gene expression patterns were analyzed across different tissues (Table S4). A total of 13,325 genes were found to be differentially expressed (DEGs) in at least one of the pairwise comparisons, including root/leaf, wood/leaf, bark/leaf, root/shoot, wood/root, wood/shoot, bark/wood, bark/root, shoot/leaf, and bark/shoot. The numbers of upregulated and down-regulated genes in each comparison are presented in Figure 3B.

To better understand the biological functions of these DEGs, KEGG and Gene Ontology (GO) enrichment analyses were performed. The KEGG pathway analysis revealed significant enrichment in pathways related to plant–pathogen interactions, phenylpropanoid biosynthesis, plant hormone signal transduction, and the biosynthesis of various plant secondary metabolites (Figure 3C). GO enrichment analysis identified representative terms, such as cellular anatomical entity, membrane, and oxidoreductase activity. Notably, 53 DEGs were specifically enriched in the paclitaxel biosynthetic process, highlighting the relevance of these genes to the targeted metabolic pathway (Figure 3D).

### 2.5. Integrated Transcriptome and Metabolome Analyses of Paclitaxel Pathways

**Figure 4:**
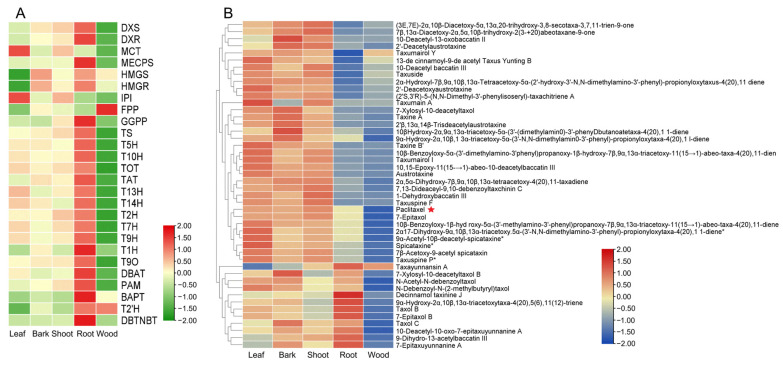
Integrated metabolomic and transcriptomic analysis of paclitaxel biosynthesis. (**A**) Differential expression of genes involved in paclitaxel biosynthesis from the five tissues. (**B**) Differential accumulation of taxoids from the five tissues.

A total of 8 upstream genes and 18 downstream genes of paclitaxel biosynthesis pathway genes were identified. Paclitaxel and several taxoids were found to significantly accumulate in the leaves, bark, and shoot. In contrast, paclitaxel derivatives, such as taxayunnansin A, decinnamoltaxinine J, and taxol B, were primarily accumulated in the root (Figure 4B). With the exception of *FPP*, *BAPT*, and *T2′H* (Figure 4A), most of the paclitaxel biosynthesis genes exhibited the lowest expression in wood. Interestingly, paclitaxel, its intermediates, and derivative metabolites correspondingly also had low levels of accumulation in wood (Figure 4B) indicating a strong correlation between gene expression and metabolite distribution across different tissues.

### 2.6. Gene Co-Expression Network Associated with Paclitaxel Biosynthesis

**Figure 5:**
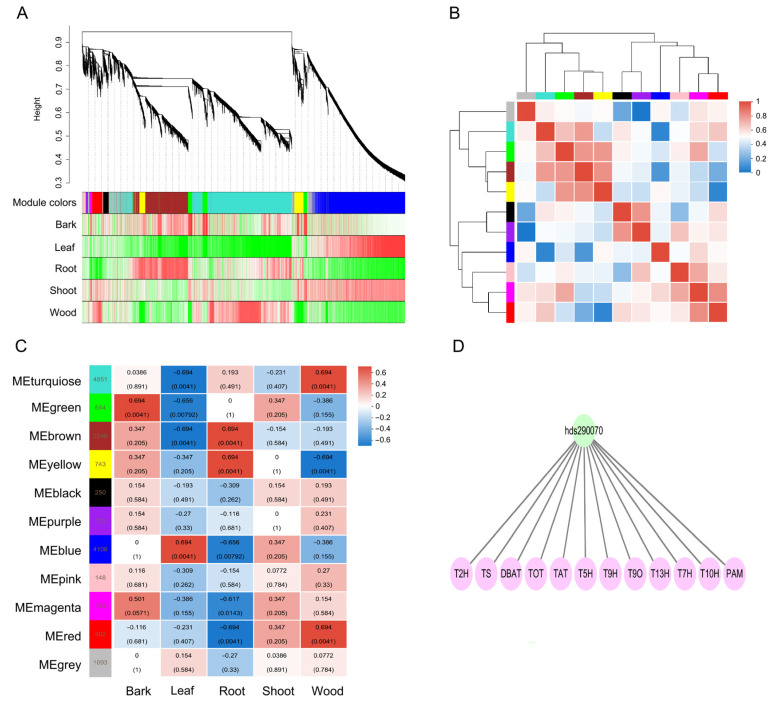
WGCNA of 13,352 DEGs. (**A**) Gene dendrogram and module colors in blocks. (**B**) Correlation between modules. (**C**) Correlations between modules and samples. (**D**) Regulatory network involved in paclitaxel biosynthesis.

To identify key TFs that might play important roles in paclitaxel biosynthesis, WGCNA was performed. Based on the expression profiles, WGCNA clustered all DEGs into 11 distinct co-expression modules (Figure 5A, Tables S5 and S6). Modules with similar expression patterns were positively correlated, whereas those with contrasting patterns were negatively correlated (Figure 5A,B). The size of these modules ranged from 4851 eigengenes (turquoise module) to 102 eigengenes (purple). The module–sample relationships showed that the yellow and brown modules were positively correlated with root (Figure 5C). Yellow, brown, and turquoise modules contained 12, 3, and 1 paclitaxel downstream biosynthesis genes, respectively (Table S7). Therefore, the yellow module was selected as the key module for further investigation into the regulatory network of paclitaxel biosynthesis. Within the yellow module, 17 TFs were identified as being co-expressed with paclitaxel biosynthesis genes, including 12 MYB, 3 ERF, and 2 bHLH (Figure S3). Interestingly, two TFs (hds060980_TmMYB3 and hds069200_bHLH46) have been previously reported to regulate paclitaxel biosynthesis [46,49], supporting the reliability of our WGCNA results.

**Figure 6:**
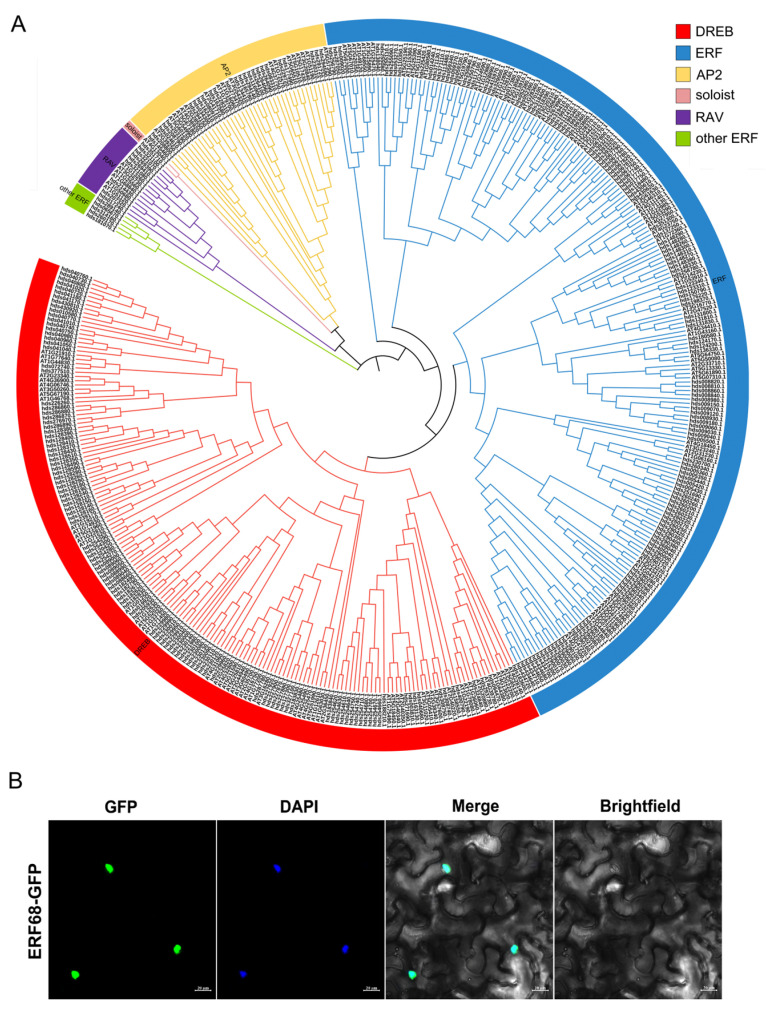
Phylogenetic analysis of AP2/ERF family genes in the *T. wallichiana* genome and subcellular localization assay of ERF68. (**A**) The phylogenetic tree of AP2/ERF transcription factors in *T. wallichiana*. (**B**) Subcellular localization of ERF68.

We identified 293 AP2/ERF family genes in the *T. wallichiana* genome (Figure 6A and Table S8). A total of 293 AP2/ERF family genes were unevenly distributed across the 12 *T. wallichiana* chromosomes (Figure S4). An ERF TF, hds290070, was renamed as ERF68 and was selected for further study (Figure 5D). To determine its subcellular localization, the GFP-fused ERF68 was transiently produced in tobacco leaves. The results show that ERF68 is a nucleus-localized protein (Figure 6B).

### 2.7. ERF68 Enhances the Expression of T2H

**Figure 7:**
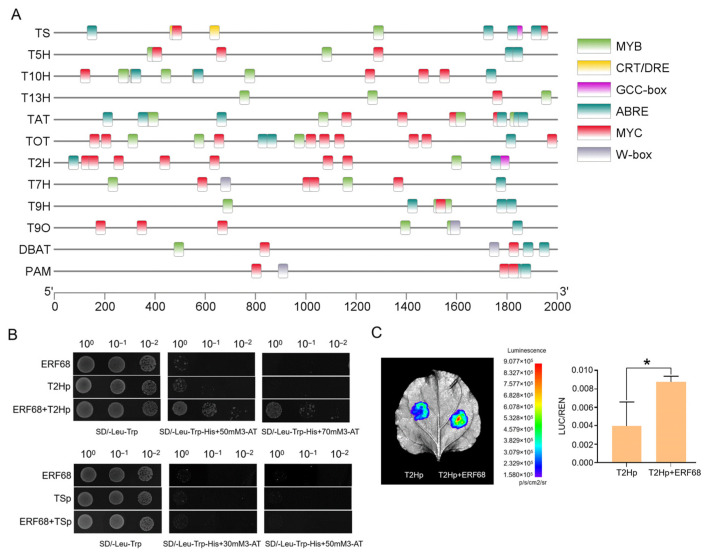
ERF68 regulates the expression of *T2H*. (**A**) Screening of TF-binding elements in the promoter regions of *TS*, *T5H*, *T13H*, *T10H*, *TAT*, *T2H*, *T7H*, *T9H*, *T9O*, *DBAT*, and *PAM* genes. (**B**) Yeast one-hybrid assay analysis of the interaction between ERF68 and the promoters of *T2H* and *TS*. (**C**) Dual-luciferase assays in tobacco leaves show that ERF68 activates the promoter activity of *T2H*. Each value is the mean ± SD of three biological repeats. “*” indicates a significant difference of *p* < 0.05.

To evaluate the role of ERF68 in *Taxus*, the promoter sequences of 12 paclitaxel biosynthesis pathway genes were analyzed (Figure 7A, Table S9). GCC-box and CRT/DRE were identified in the promoter of *TS*. GCC-box was identified in the promoter of *T2H*. The Y1H assays confirmed the interaction of ERF68 and *T2H*, indicating that ERF68 may regulate the expression of *T2H* by directly binding its promoter (Figure 7B). Furthermore, dual-luciferase reporter assays in tobacco leaves showed that ERF68 significantly activated *T2H* expression (Figure 7C).

## 3. Discussion

Paclitaxel, a well-known anticancer agent, inhibits cancer cell proliferation by stabilizing microtubules [5]. Since its approval for clinical application by the U.S. Food and Drug Administration (FDA), extensive research has been devoted to elucidating its biosynthetic and metabolic regulation in *Taxus* species [2,51]. With the recent release of *Taxus* genome sequences, the biosynthetic pathway of paclitaxel has been largely deciphered [13,14,15]. Transcriptome profiling was conducted for different tissues of *Taxus* [14,15,52,53,54]. However, the tissue-specific accumulation patterns of paclitaxel and related taxoids in *Taxus* remain incompletely understood [52,53,54].

Over the past 60 years, nearly 600 taxoids—including paclitaxel, cephalomannine, 10-deacetylbaccatin III (10-DAB), baccatin III, and others—have been isolated from the *Taxus* genus [36]. Previous studies have reported differences in the accumulation of paclitaxel and taxoids between different stem tissues. Paclitaxel and 10-DAB were mainly accumulated in the phloem and bark, whereas baccatin III showed high accumulated in wood tissues [46,55]. In this study, we employed an integrative approach combining widely targeted metabolomics and transcriptomics to investigate paclitaxel biosynthesis and tissue-specific accumulation across five major tissue types—leaf, shoot, bark, wood, and root—of *T. chinensis* var. *mairei*. The resulting datasets provide a valuable resource for analyzing the tissue distribution patterns of taxiods and identifying key transcription factors regulating their biosynthesis. This can help to elucidate secondary metabolite regulatory networks and guide breeding strategies aimed at enhancing metabolite content in *Taxus*.

Using a UPLC-MS/MS-based widely targeted metabolomics approach, we identified 1616 DAMs that were differential accumulated in at least two tissues. These DAMs were grouped into two major clusters, each representing distinct tissue accumulation patterns. Cluster I (mainly enriched in the root, wood, and bark) was involved in terpenoid backbone biosynthesis, while Cluster II (enriched in the leaf, shoot, and bark) was associated with diterpenoid biosynthesis. This suggests that paclitaxel biosynthesis may mainly synthesize in the root or wood, and that its intermediates or end products may be transported to the leaf, shoot, and bark tissues. Referring to the *T. wallichiana* genome [14], the transcriptional difference of five tissues was analyzed. A total of 13,325 DEGs were identified across tissue comparisons. KEGG and GO enrichment analyses revealed a significant involvement of DEGs in secondary-metabolite biosynthesis pathways, including the paclitaxel biosynthetic process. Most paclitaxel biosynthetic genes showed a high expression in the root, yet paclitaxel and major taxoids were highly accumulated in the leaf, shoot, and bark, while paclitaxel derivatives, such as decinnamoltaxinine J, taxol B, and 7-epitaxol B, were highly accumulated in the roots. The discrepancy between gene expression and metabolite accumulation implies the transport of taxoids across tissues, potentially as a mechanism to mitigate cytotoxicity by relocating paclitaxel and related compounds from the roots to aerial tissues [46,48,55].

Previous studies have identified transcription factors from the MYB, ERF, WRKY, and bHLH families as regulators of paclitaxel biosynthesis [41,42,43,44,45,46,47,48,49]. In this study, we constructed a transcriptional regulatory network using WGCNA. This network not only validated previously reported regulators, but also identified several novel transcription factors potentially involved in paclitaxel biosynthesis. For instance, earlier studies reported that the MYB transcription factor TmMYB3 regulates *TS* and *TBT* expression in *T. media* [46]. In our dataset, we confirmed the co-expression between the homolog of *TmMYB3* (*hds060980*) and *TS*, further validating the reliability of our data. Moreover, 15 previously unreported TFs were implicated in regulating paclitaxel biosynthesis. Sun et al. reported a regulatory network, including 10 enzyme genes of the paclitaxel pathway and 28 TFs [50]. The regulatory network we constructed involved 12 genes of the paclitaxel pathway, which was more than that reported by Sun et al. Notably, we confirmed the regulatory role of ERF68 on *T2H* expression using yeast one-hybrid (Y1H) assays and dual-luciferase (LUC) analysis. To date, only two ERF TFs—TcERF12 and TcERF15—have been functionally characterized in paclitaxel biosynthesis regulation, acting as a negative and a positive regulator of *TS* [36]. The activation effect of our ERF68 on the paclitaxel biosynthesis gene *T2H* revealed a new mechanism underlying the transcriptional regulation of paclitaxel biosynthesis.

## 4. Materials and Methods

### 4.1. Plant Materials and Growth Conditions

Five-year-old *T. chinensis* var. *mairei* seedlings were grown in the greenhouse of the Chinese Academy of Forestry. The leaves, shoots, barks, woods, and roots were collected and immediately flash-frozen in liquid nitrogen from three *T. mairei* plants with a similar growth status. All samples were stored at −80 °C for subsequent metabolomic analysis and transcriptomic analysis.

### 4.2. Metabolite Extraction and Detection

Metabolite extraction and detection were provided by Wuhan Maiwei Metabolic Biotechnology Co., Ltd. (Wuhan, China). The tissue samples were freeze-dried and ground into a fine powder. A total of 50 mg of powder was extracted with 1.2 mL of 70% aqueous methanol. The mixture was vortexed every 30 min for 30 s, 6 times in total, and then centrifuged at 12,000 rpm for 3 min. Supernatant was collected and filtered through a microporous filtration membrane (0.22 µm pore size) for ultra-performance liquid chromatography tandem mass spectrometry (UPLC−MS/MS) analysis.

The composition of the extracts was determined using ultra-performance liquid chromatography coupled with tandem mass spectrometry. The liquid chromatography column was Agilent (Santa Clara, CA, USA) SB-C18 1.8 µm, 2.1 mm × 100 mm. The mobile phase consisted of a 0.1% aqueous solution of formic acid (A) and a solution of acetonitrile (B). The gradient program was set according to the following conditions: 0 min, 5% of solvent B; 0~9 min, 5 to 95% of solvent B; 9~10 min, 95% of solvent B; 10~11 min, 5% of solvent B; and 11~14 min, 5% of solvent B. The flow rate was maintained at 0.35 mL/min, the column temperature was set at 40 °C, and the injection volume was 2 μL. MS/MS analysis conditions were as follows: electrospray ionization temperature was set at 500 °C; the ion spray voltages were set at 5500 V (positive ion mode) and 4500 V (negative ion mode); the ion source gas I, gas II, and curtain gas were set to 50, 60, and 25 psi, respectively; and the collision-induced ionization parameter was set high. The triple-quadrupole (QQQ) scans were performed in the multiple-reaction monitoring (MRM) mode with collision gas (nitrogen) set to medium. The declustering potential (DP) and collision energy (CE) for individual MRM transitions were performed based on the optimized DP and CE. A specific set of MRM transitions was monitored for each period according to the metabolites eluted during this period.

### 4.3. Analysis of Widely Targeted Metabolomic Data

PCA was implemented using the stats package [56] in R (version 4.1.2) after data preprocessing was performed using unit variance scaling (UV) normalization. The cluster heatmap was created using Metware Cloud (https://cloud.metware.cn) (accessed on 11 January 2025). OPLS-DA was generate using the OPLSR Anal function in the R package MetaboAnalystR (version 1.0.1) [57]. Data preprocessing was performed using logarithmic normalization and zero-centered (Ctr) before performing OPLS-DA. A one-way analysis of variance (ANOVA) was carried out to compare the content differences of metabolites between pairwise comparisons (root/leaf, wood/leaf, bark/leaf, root/shoot, wood/root, wood/shoot, bark/wood, bark/root, shoot/leaf, and bark/shoot) using R (version 3.5.1). Multivariate statistical analysis with threshold values of VIP ≥ 1 (VIP based on OPLS-DA) and a *p*-value < 0.05 was performed to analyze tissue DAMs. The relative content of all DAMs was pretreated with UV, and then K-means clustering analysis was performed using R (version 3.6.2). The metabolites were annotated using the KEGG database (http://www.kegg.jp/kegg/compound/) (accessed on 8 July 2024) and mapped to the KEGG pathway database (http://www.kegg.jp/kegg/pathway.html) (accessed on 8 July 2024).

### 4.4. RNA Extraction, Library Construction, and RNA-Seq

Total RNA was extracted from five tissues (leaves, shoot, bark, wood, and root) of *T. chinensis* var. *mairei* with a total RNA extraction kit (Majorbio, Shanghai, China). Purity and quantity of RNA were assessed by the 5300 Bioanalyser (Agilent Technologies, Santa Clara, CA, USA) and Nano Drop 2000 spectrophotometer (Thermo Scientific, Waltham, MA, USA). The sequencing library was constructed following illumina**^®^** Stranded mRNA Prep, Ligation (illumina, San Diego, CA, USA) using 1 µg of RNA from each sample. RNA sequencing was performed on the Nova Seq × Plus platform (illumina, San Diego, CA, USA). The raw paired-end reads were trimmed and quality-controlled by fastp (https://github.com/OpenGene/fastp) (accessed on 18 September 2024) with default parameters. The clean reads were mapped to the *T. wallichiana* reference genome [14] on HISAT2 version 2.2.1 [58].

### 4.5. Differential Expression Analysis and Functional Enrichment

PCA was conducted on the online tool Majorbio Cloud Platform (https://cloud.majorbio.com/page/tools/) (accessed on 18 September 2024). To identify DEGs between different tissues, the expression level of each transcript was quantified according to the read counts. RSEM [59] was used to quantify gene abundances. Essentially, differential expression analysis was performed using DESeq2 (Version 1.42.0) [60]. The BH method (Benjamini–Hochberg, which is the same as FDR) was used for multiple testing correction. And the genes with |log2FC| ≧ 1 and FDR < 0.05 were identified DEGs. In addition, GO and KEGG functional enrichment analyses were subsequently carried out by Goatools (Version 1.4.4) [61] and Python scipy software (Version1.0.0), respectively.

### 4.6. WGCNA Analysis

WGCNA analysis was performed on the online tool Majorbio Cloud Platform (https://cloud.majorbio.com/page/tools/) (accessed on 30 October 2024) with the TPM of DEGs as the input [62,63]. The co-expression modules were obtained using the automatic network construction function (blockwise Modules) with a soft threshold power of 5, network type was signed, merge CutHeight was 0.25, and min module size was 500. Eigengene values were calculated for each module based on the Spearman’s correlation. The weighted value of the correlation coefficient was used in the WGCNA analysis, which made the connections between genes in the network obey scale-free networks. The hierarchical clustering tree was constructed according to the correlation coefficient between genes. Genes with similar expression patterns were grouped into the same module. The module containing paclitaxel biosynthesis genes was selected for analysis. The networks were visualized by Cytoscape software (version 3.7.1).

### 4.7. Identification of the AP2/ERF Family Member

The hidden Markov model (HMM) of the AP2 domain (PF00847) obtained from the Pfam database (http://pfam.xfam.org/) (accessed on 26 May 2024) was used to conduct the putative AP2 proteins search in *T. wallichiana* by Tbtoolsv1.098696 software. And the *Arabidopsis thaliana* AP2/ERF protein sequences were downloaded from the PlantTFDB database and used as a query to search against AP2/ERF candidate proteins in *T. wallichiana* by BLASTp. All putative AP2/ERF proteins were further confirmed using PlantTFDB, SMART (http://smart.embl.de/) (accessed on 24 April 2025), and InterPro tools (http://pfam.xfam.org/) (accessed on 16 April 2025). A phylogenetic tree was constructed by MEGA7 software [64] using the Neighbor-Joining (NJ) method with 1000 bootstraps. Then, the phylogenetic tree was colored by the online website of iTOL (https://itol.embl.de/) (accessed on 23 April 2025). All identifiedAP2/ERF genes were mapped to the *T. wallichiana* chromosomes using Tbtoolsv1.098696 software.

### 4.8. Subcellular Localization

The open reading frame of ERF68 was cloned and inserted into the pCAMBIA1300 vector, generating 35S-ERF68:GFP. 35S-ERF68:GFP and 35S:GFP were separately transiently expressed in *Nicotiana benthamiana* leaves via *Agrobacterium tumefaciens* strain GV3101. After 48–72 h, the infiltrated leaves were collected and incubated with a phosphate-buffered saline containing 4′,6′-diamidino-2-phenylindole (DAPI). The fluorescence signals were detected using a confocal microscope (LSM 880, Zeiss, Baden Wurttemberg, Germany). The primers used for subcellular location are provided in Table S10.

### 4.9. Promoter Isolation and Cis-Element Scanning

Genomic DNA was isolated from *T. mairei* root using the classical cetyl trimethyl ammonium bromide method. The 2000 bp promoter sequences of *TS*, *T2H*, *T5H*, *T7H*, *T9H*, *T10H*, *T13H*, *TAT*, *T9O*, *DBAT*, *PAM*, and *TOT* were extracted according to the *T. mairei* genome [14]. Then, the promoter sequences were uploaded and scanned using the PlantCARE program (http://bioinformatics.psb.ugent.be/webtools/plantcare/html/) (accessed on 29 March 2025) and visualized using TBtools-II (Toolbox for Biologists) v2.152 software.

### 4.10. Yeast One-Hybrid Assays

The promoters of *T2H* and *TS* were cloned and inserted into the PHIS2 vector, respectively. The coding region of *ERF68* was inserted into the pGADT7 vector. The resulting vector was co-transformed into a yeast Y187 cell and grown on SD/-Leu/-Trp medium. The activation activity was examined on the SD/-Leu/-Trp/-His/-3AT medium. The primers are shown in Table S10.

### 4.11. Dual-Luciferase Reporter Assay

The promoter of *T2H* was inserted into the pGreen 0800-LUC reporter vector. The coding region of *ERF68* was cloned into the pGreenII 62-SK effector vector. The empty pGreenII 62-SK effector vector were used as a control. All completed constructs were co-transformed into *N. benthamiana* leaves by *A. tumefaciens* (GV3101)-mediated transient expression. Detection of Firefly Luciferase (LUC) and Renilla (REN) luciferase activities was performed using a Dual-Luciferase**^®^** Reporter Assay System (Promega, Madison, WI, USA). The ratio of LUC to REN was used to analyze the results, and 3 biological repeats were set. The primers used for the dual-luciferase reporter assay are shown in Table S10.

## 5. Conclusions

In summary, we integrated metabolomic and transcriptomic approaches to investigate the biosynthesis and accumulation patterns of paclitaxel across five major tissue types (leaf, shoot, bark, wood, and root) in *T. mairei*. Most enzyme genes involved in paclitaxel biosynthesis showed the highest expression in the root. Paclitaxel was significantly enriched in the leaf, bark, and shoot. Paclitaxel derivatives, such as taxayunnansin A and taxol B, accumulated primarily in the root. We constructed a regulatory network associated with paclitaxel biosynthesis. Furthermore, ERF68 was identified as a potential positive regulator of paclitaxel biosynthesis. These findings offer valuable insights into the regulatory mechanisms underlying paclitaxel production and provide a foundation for future efforts in the genetic improvement and metabolic engineering of *Taxus* species.

## Data Availability

All data supporting the findings of this study are included in the article/Supplementary Materials.

## Supplementary Materials

The following supporting information can be downloaded at: https://www.mdpi.com/article/10.3390/plants14121775/s1, Figure S1. The total ion chromatograms (TICs) of the mixed-sample extracts; Figure S2. The number of genes annotated by different databases, including GO, KEGG, EggNOG, NR, Swiss-Prot, and Pfam; Figure S3. Regulatory network involved in paclitaxel biosynthesis; Figure S4. The distribution of AP2/ERF genes on 12 *T. wallichiana* chromosomes; Table S1. The detailed information of metabolite annotations; Table S2. The detailed information of the RNA-sequencing; Table S3. Mapping rates for each sample; Table S4. The detailed information of all genes; Table S5. Members in modules; Table S6. The connectivity of co-expressed genes in different modules; Table S7. Modules of paclitaxel biosynthesis genes; Table S8. The ERF family in *T. wallichiana*; Table S9. The promoter sequences of the paclitaxel biosynthesis genes; Table S10. The primer sequences used in the present study.
