# Supplementary figures and images for: Transcriptome and Metabolome Analyses of Taxus chinensis var. mairei Tissues Provide New Insights into the Regulation of Paclitaxel Biosynthesis
Source: Plants (Basel). 2025 Jun 10;14(12):1775. doi: 10.3390/plants14121775 (PMC12196951; doi:10.3390/plants14121775)

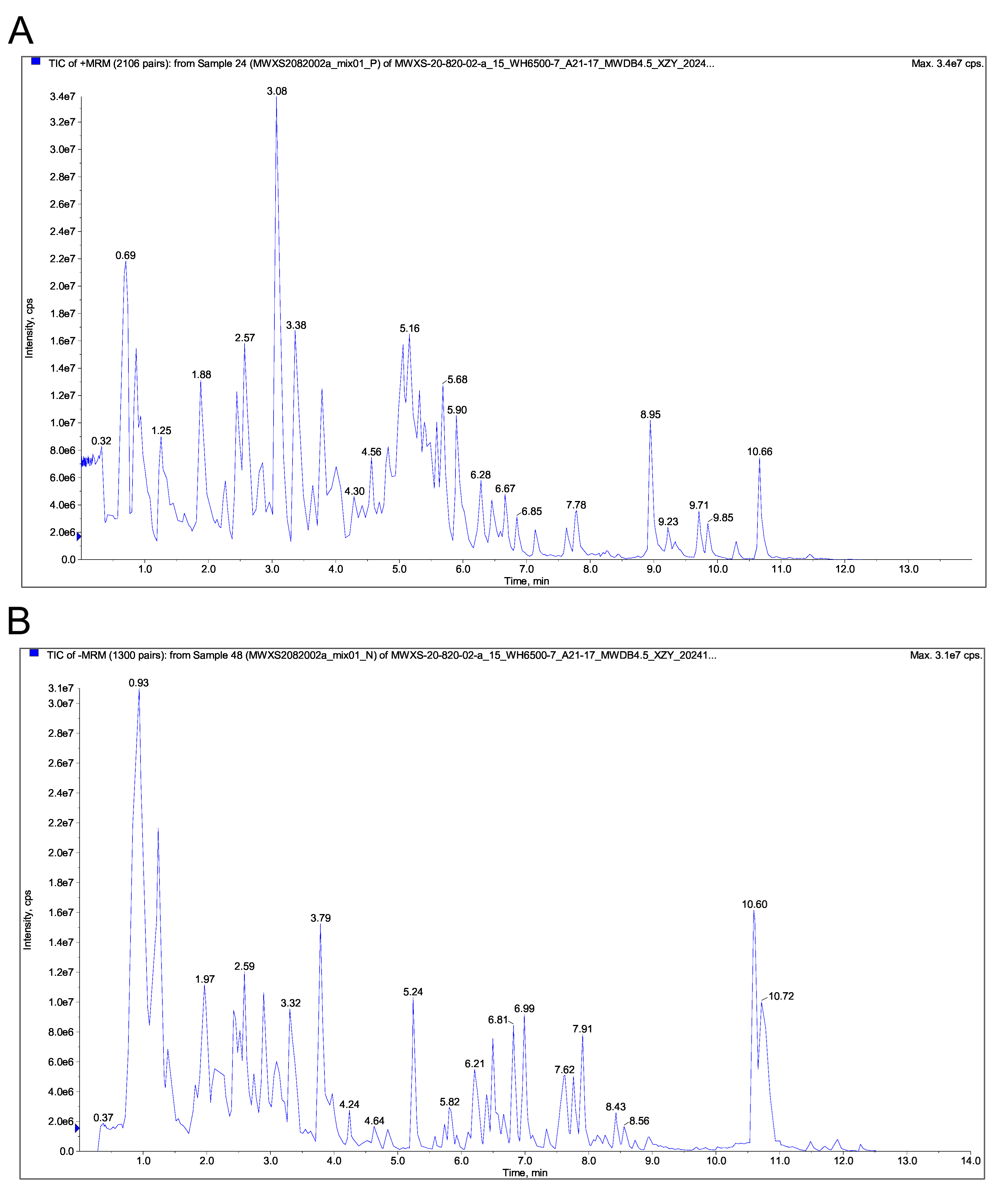

Supplement: Supplementary file 1 [file plants-14-01775-s001.zip › Figure S1.tif]

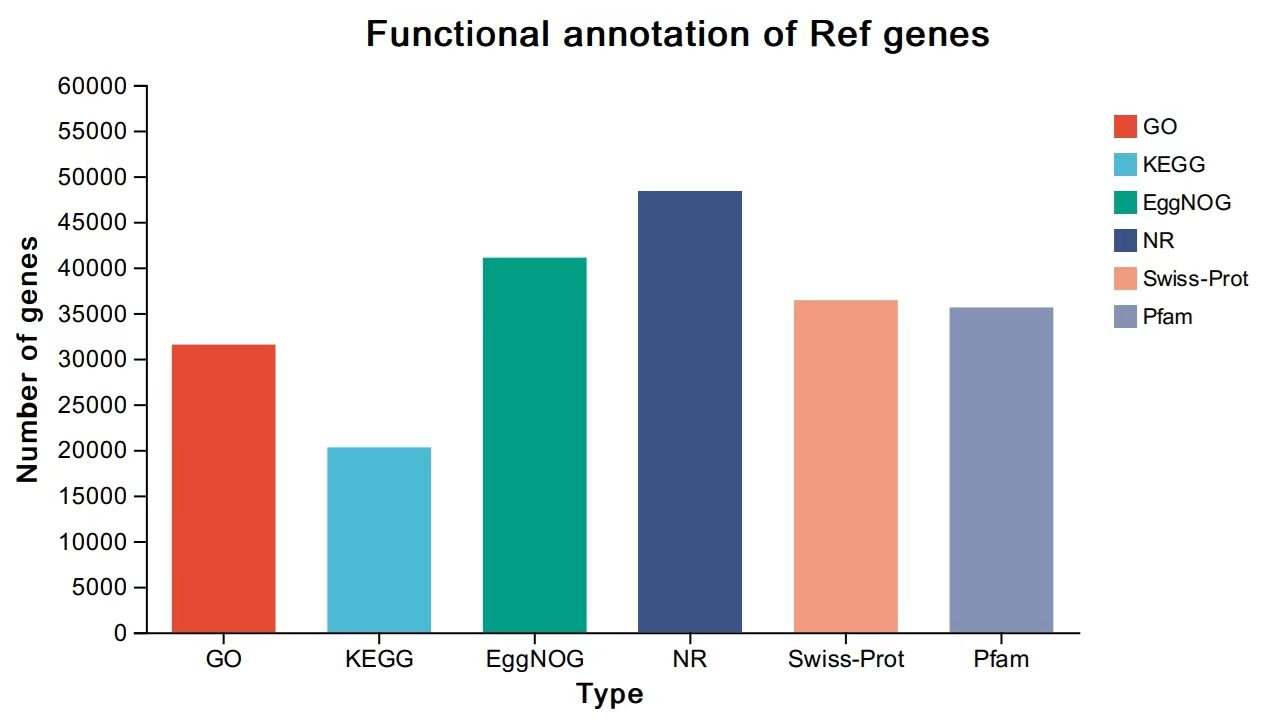

Supplement: Supplementary file 1 [file plants-14-01775-s001.zip › Figure S2.tif]

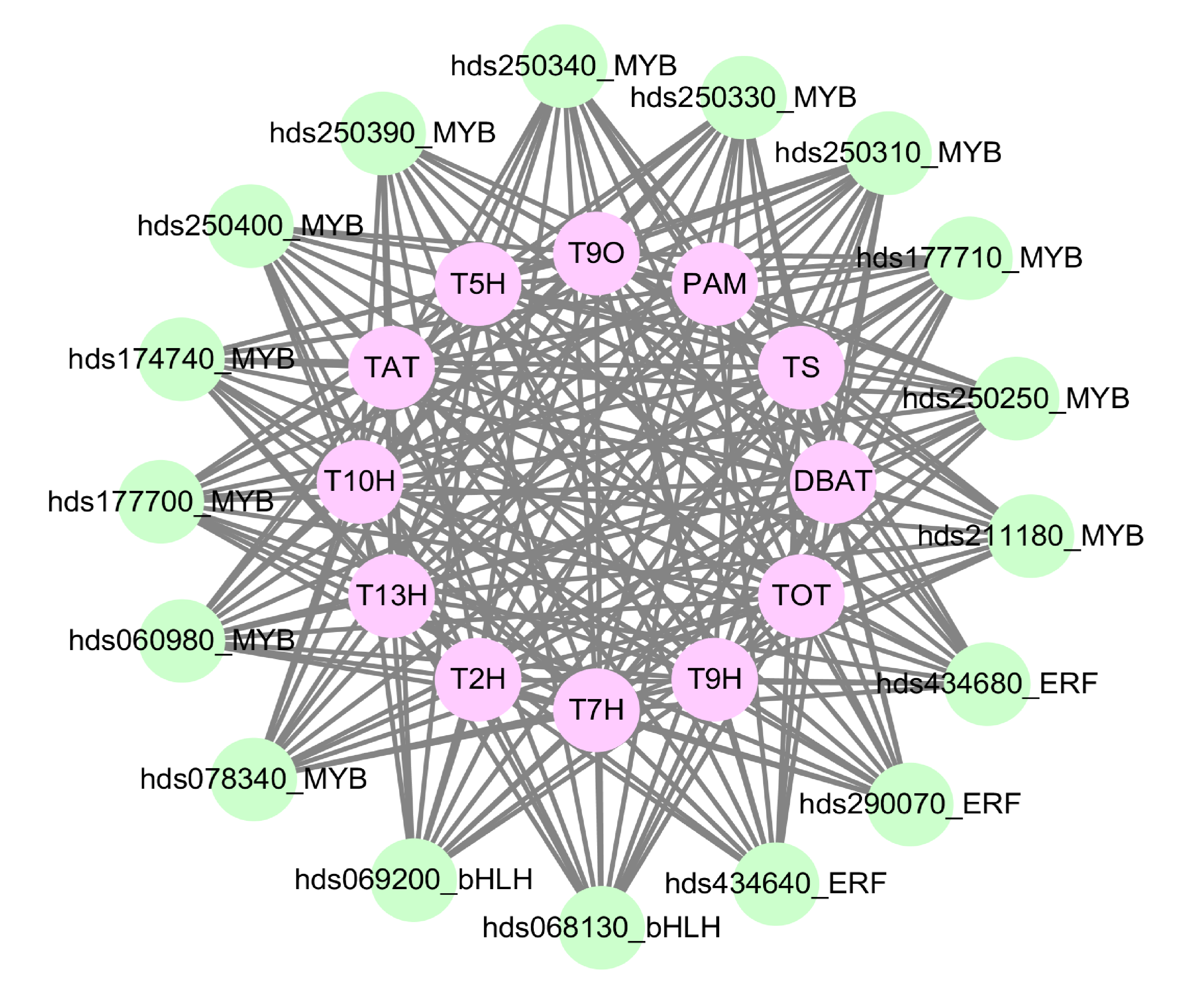

Supplement: Supplementary file 1 [file plants-14-01775-s001.zip › Figure S3.tif]

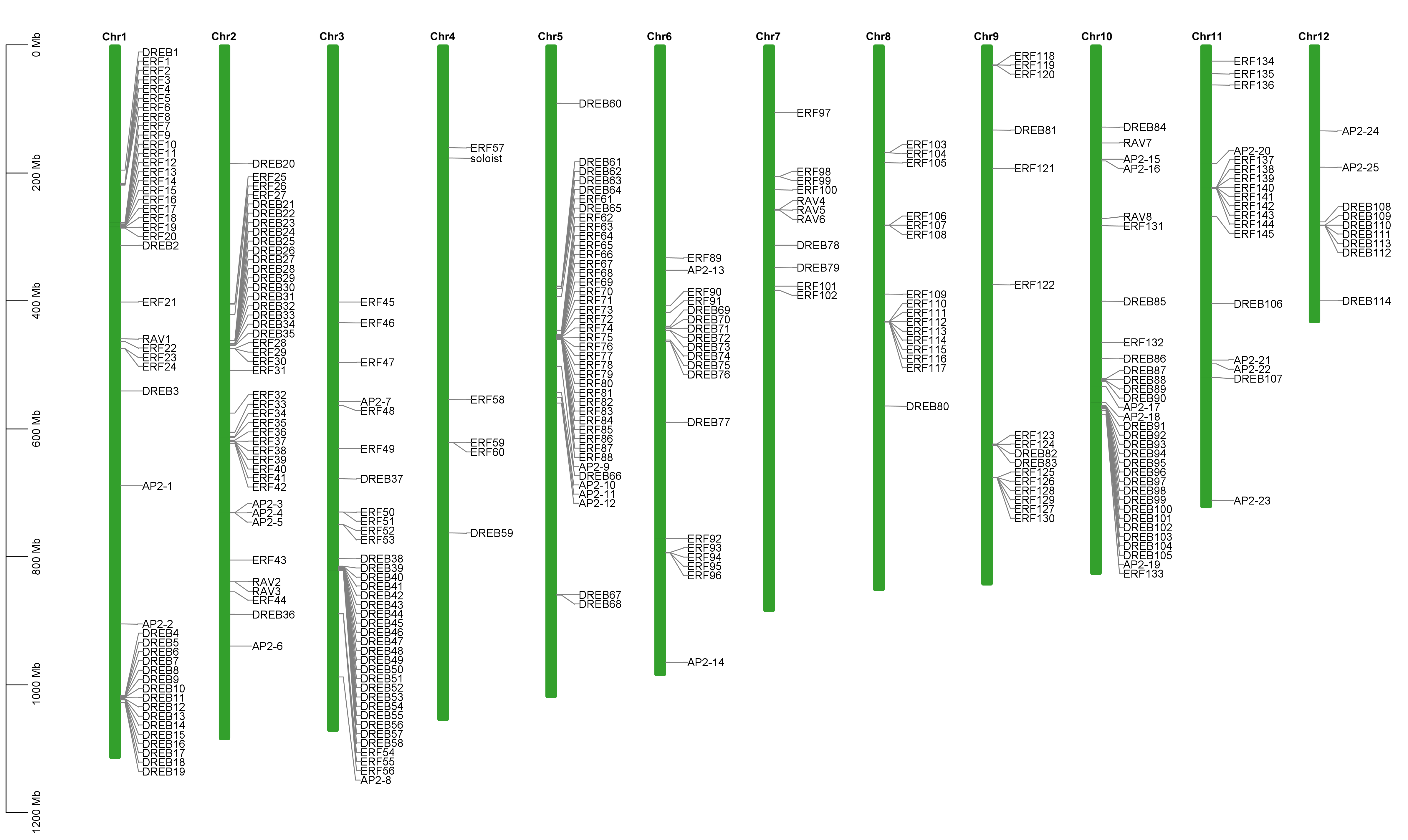

Supplement: Supplementary file 1 [file plants-14-01775-s001.zip › Figure S4.tiff]
